# Retrospective Review of Flexible Bronchoscopy in Pediatric Cancer Patients

**DOI:** 10.3389/fonc.2021.770523

**Published:** 2021-12-14

**Authors:** Ali H. Ahmad, Brandon D. Brown, Clark R. Andersen, Kris M. Mahadeo, Demetrios Petropolous, José A. Cortes, Shehla Razvi, Mary Katherine Gardner, Linette J. Ewing, Rodrigo E. Mejia

**Affiliations:** ^1^ Pediatric Critical Care Medicine, The University of Texas MD Anderson Cancer Center, Houston, TX, United States; ^2^ Pediatric Oncology Fellowship Program, The University of Texas MD Anderson Cancer Center, Houston, TX, United States; ^3^ Biostatistics, The University of Texas MD Anderson Cancer Center, Houston, TX, United States; ^4^ Pediatric Stem Cell Transplantation and Cellular Therapy and CARTOX Program, The University of Texas MD Anderson Cancer Center, Houston, TX, United States

**Keywords:** bronchoscopy, BAL, pediatric oncology, pediatric critical care, pediatric cancer

## Abstract

The use of flexible bronchoscopy (FB) with bronchoalveolar lavage (BAL) to diagnose and manage pulmonary complications has been shown to be safe in adult cancer patients, but whether its use is safe in pediatric cancer patients remains unclear. Thus, to describe the landscape of FB outcomes in pediatric cancer patients and to help define the populations most likely to benefit from the procedure, we undertook a retrospective review of FBs performed in patients younger than 21 years treated at our institution from 2002 to 2017. We found that a greater volume of total fluid instilled during BAL was significantly associated with increased probabilities of positive BAL culture (p=0.042), positive bacterial BAL culture (p=0.037), and positive viral BAL culture (p=0.0496). In more than half of the FB cases, findings resulted in alterations in antimicrobial treatment. Our study suggests that for pediatric cancer patients, FB is safe, likely provides diagnostic and/or therapeutic benefits, and has implications for treatment decisions.

## Introduction

Pulmonary complications are common events that result in significant morbidity and mortality for cancer patients of all ages (1–4). The presence of pulmonary infiltrates in cancer patients presents a diagnostic dilemma. The differential diagnosis includes malignant disease, inflammatory response, and infectious disease, the last of which could have a bacterial, viral, or fungal etiology. Noninvasive imaging is of limited utility in differentiating among these diagnoses. Thus, clinicians must often choose among invasive procedures, including flexible bronchoscopy (FB) with bronchoalveolar lavage (BAL), endobronchial ultrasound (EBUS) (5), radial EBUS, robotic bronchoscopy and computed tomography (CT) guided navigation (6), needle aspiration, and open lung biopsy, to yield a diagnosis. Of these options, FB with BAL is uniquely useful as a diagnostic and therapeutic lung examination procedure. Moreover, the BAL fluid obtained during FB provides a sample of the alveolar microenvironment, reflecting the greater respiratory system. This fluid may yield microbes such as bacteria, inflammatory cells such as macrophages, or malignant cells such as leukemic cells, which, if diagnostic, can guide the management of cancer patients with pulmonary infiltrates (7, 8).

In the present study, our primary objective was to describe the diagnostic yield of FB with BAL in pediatric (9) cancer patients. Our secondary objectives were to identify patient demographics that influence the diagnostic yield; determine the safety of FB and describe its short-, medium-, and long-term outcomes; and describe the rate of patients in whom FB results changed clinical management.

## Methods

### Patients

We conducted a retrospective chart review of all cancer patients younger than 21 years who underwent FB at MD Anderson from 2002 to 2017. Patients were identified from a database obtained from an electronic medical record query for FBs performed on pediatric patients by different physicians. We obtained discrete data, including cancer diagnosis; age; sex; laboratory values; diagnostic imaging data; pathology and microbiology results; lung biopsy results; antimicrobial treatment at the time of FB and after FB; noninvasive and/or mechanical ventilation data; indication for FB; location of FB; type of airway device or equipment used during bronchoscopy if indicated; specialty of the physician performing FB; number of BALs; volume of BAL fluid instilled; BAL return yield; lung segment and laterality of BAL; complications of FB; type and timing of HSCT; duration of hospitalization and intensive care unit (ICU) stay; and mortality. Patients received platelet transfusions prior to BAL if the platelet count was < 50 K/µL. Patients with a history refractory thrombocytopenia received a platelet transfusion at the time of the FB. If bleeding occurred during FB, patients were given additional platelet transfusions as clinically appropriate. Clotting parameters were requested only for patients with bleeding tendencies prior to the FB.

### Informed Consent and Data Security

MD Anderson’s Institutional Review Board approved the study and waived the requirement for informed consent. Protected health information was initially collected, but names and medical record numbers were replaced with study numbers in the analytical file and were not published nor part of the aggregate data. Procedure and treatment dates were retained for analysis.

Information was retained on a password-protected network server. Protocol-specific study numbers were created for each study participant, and collected data were maintained in a secure password-protected database located on a departmental network server housed behind MD Anderson’s firewall. Complete confidentiality was maintained throughout the study and the preparation and submission of the manuscript.

### Statistical Analysis

Patient demographics and clinical characteristics were described and analyzed using means and medians for continuous variables and percentages for categorical variables.

Baseline variables were summarized as means with standard deviations or as frequencies with percentages. Rates of overall survival (calculated from the time of FB) at 3, 28 and 180 days were analyzed using the Kaplan-Meier method (10).

Binary outcomes were modeled by mixed effect logistic regression with relation to each continuous or discrete covariate, with a subject block to control for repeated measures. Continuous outcomes were modeled by mixed-effect linear regression with relation to each continuous or discrete covariate. Where appropriate, these linear models were replaced with generalized additive mixed-effect models with penalized splines accommodating nonlinear relations between the outcome and continuous covariates. Model-adjusted differences in outcomes among levels of discrete covariates were estimated with Tukey-adjusted contrasts.

Statistical analyses were performed using R statistical software (11). All statistical tests were two-sided, with alpha=.05. Survival modeling was performed using the “survival” package (12, 13). Differences among discrete variable levels of covariates in the linear and logistic models were estimated using the “emmeans” package; this analysis included adjusted means weighted proportionally to the covariate marginal frequencies. Catseye plots were produced using the “catseyes” package (14–16).

## Results

### FB Characteristics

Patient demographics are summarized in **Table 1**. FB characteristics are summarized in **Table 2**. During the 16-year study period, 198 pediatric cancer patients underwent a total of 264 fiberoptic FBs (range, 1-6 FBs per patient). The mean patient age at the time of FB was 15.5 ± 5.2 years (range: 1.6-21.0 years). The most common indication for FB was BAL, in 196 (99.0%) patients.

**Table 1:** Patient demographics.

Characteristic	No. of patients (%), *n = 198
Mean patient age ± SD, years	15 ± 5.2
Female patients	85 (42.9)
Oncologic diagnosis	
ALL	61 (30.8)
AML	54 (27.3)
Hodgkin lymphoma	30 (15.1)
Other diagnosis	53 (26.8)
Transplant status	
Non-transplant	123 (62.1)
HSCT	75 (37.9)
Autologous	10
Allogeneic cord	30
Allogeneic related-donor	12
Allogeneic unrelated-donor	19
Haploidentical	4

SD, standard deviation; ALL, acute lymphoblastic leukemia; AML, acute myeloid leukemia; HSCT, hematopoietic stem cell transplantation.

*Unless otherwise noted.

**Table 2:** Flexible bronchoscopy (FB) characteristics.

Characteristic	No. of FBs (%), *n = 264
Indication for FB	
BAL	245 (92.8)
Atelectasis	3 (1.1)
ETT position	1 (0.4)
Bronchial washing	1 (0.4)
Hypercapnia	3 (1.1)
Airway obstruction	1 (0.4)
Diagnostics	8 (3.0)
BAL + transbronchial biopsy	2 (0.8)
Bronchoscope insertion route	
Oro/nasopharynx	105 (39.8)
LMA	35 (13.3)
ETT	113 (42.8)
Tracheostomy	11 (4.2)
Mean patient ± SD, BAL volume (mL)	
Children (1-11 years)	46 ± 28
Adolescents (12-21 years)	76 ± 32
Antimicrobial treatment	
Empiric antimicrobial treatment	233 (88.3)
Gram-positive antibacterial treatment	173 (65.5)
Gram-negative antibacterial treatment	193 (73.1)
Atypical antibacterial treatment	82 (31.1)
Anaerobic antibacterial treatment	19 (7.2)
Antiviral treatment	107 (40.5)
Antifungal treatment	180 (68.1)
PJP treatment	69 (26.1)
Acute respiratory failure	95 (36.0)

BAL, bronchoalveolar lavage; LMA, laryngeal mask airway; ETT, endotracheal tube; mL, milliliters; PJP, Pneumocystis jirovecii.

*Unless otherwise noted.

The most common underlying oncologic diagnoses were acute lymphoblastic leukemia in 61 (30.8%) patients, acute myelogenous leukemia in 54 (27.3%) patients, and Hodgkin lymphoma in 30 (15.2%) patients. Of the 198 patients included in the study, 75 (37.9%) underwent at least one HSCT.

At least one empiric antimicrobial treatment was initiated before FB in 233 (88.3%) cases. Ninety-five (36.0%) FBs were performed in patients who had acute respiratory failure and were on mechanical ventilation. Thirty-eight (14.4%) FBs were performed in patients who had acute respiratory failure and were on invasive mechanical ventilation with a positive end-expiratory pressure (PEEP) ≥10 cmH_2_0 and a fraction of inspired oxygen (FiO_2_) ≥0.5.

### Complications

FB-related complications occurred in 26 (9.8%) cases. In 10 (3.8%) cases, FB-related complications occurred in patients who had acute respiratory failure and were on invasive mechanical ventilation; in 4 (1.5%) cases, the patients had complications while receiving ventilation with a PEEP ≥ 10 cmH_2_0 and a FiO_2_ ≥ 0.5. Major complications including hypoxia, tachycardia, hypotension and altered mental status occurred in 10 patients (3.8%). Minor complications included transient hypoxia and/or bronchospasm, bleeding and other occurred in 16 patients (6%) (**Table 3**). The most common major complication was hypoxia in 6 cases (2.3%), requiring ICU admission for non-invasive or invasive mechanical ventilation, with one (0.4%) patient requiring hospital transfer for inhaled nitric oxide. The most common minor complication was self limited bleeding in 5 (1.9%) cases with one patient (0.4%) requiring instillation of epinephrine. No associated mortality occurred as a result of FB.

**Table 3:** Complications.

Characteristic	Children (1-11 years)	Adolescents (12-21 years)
	(n)	(n)
Major complications		
Hypoxia	4	4
Tachycardia	1	
Hypotension		1
Altered mental status		2
Minor complications		
Transient hypoxia	1	2
Hypoxia and Bronchospasm	3	
Bleeding		6
Other		2

### Clinical Data

Clinical data are summarized in **Table 4**. These are categorized as respiratory function, which details mean ventilator settings and mean PaO_2_/FiO_2_ ratio, and bone marrow function, which details mean results of Complete Blood Count (CBC) with differential laboratory data

**Table 4:** Clinical data.

Variable	Mean ± SD*
Respiratory function	
SpO_2_, %, n=93	97.10 ± 2.60
Blood gas results	
PaO_2_, mmHg, n=82	128.30 ± 70.30
PaCO_2_, mmHg, n=90	44.80 ± 10.20
Ventilator settings	
PIP, cmH_2_O, n=54	28.4 ± 7.00
PEEP, cmH_2_O, n=84	9.6 ± 2.50
FiO_2_, n=93	0.61 ± 0.23
P/F ratio, n=82	224.50 ± 115.0
S/F ratio, n=88	181.90 ± 66.70
Bone marrow function	
WBC count, leukocytes/mL, n=263	6,000 ± 9,100
ANC, neutrophils/mL, n=210	5,000 ± 6,900
AMC, monocytes/mL, n=197	800 ± 2,300
ALC, lymphocytes/mL, n=206	900 ± 1,700
Hemoglobin level, g/dL, n=260	9.5 ± 1.5
Platelet count, K/µL	102.8 ± 114.5
No. of FBs in patients with leukopenia (%)	
Mild neutropenia (ANC <1500/mL)	63 (23.9)
Severe neutropenia (ANC <500/mL)	34 (12.9)
Monocytopenia (AMC <100/mL)	43 (16.3)

SD, standard deviation; SpO_2_, blood oxygen saturation; PaO_2_, arterial blood partial pressure of oxygen; PaCO_2_, arterial blood partial pressure of carbon dioxide; PIP, peak inspiratory pressure; PEEP, positive end-expiratory pressure; FiO_2_, fraction of inspired oxygen; P/F ratio, measured partial pressure of oxygen in blood to fraction of inspired oxygen; S/F ratio, blood oxygen saturation to fraction of inspired oxygen; WBC, white blood cell; ANC, absolute neutrophil count; AMC, absolute monocyte count; ALC, absolute lymphocyte count; FB, flexible bronchoscopy.

*Unless otherwise noted.

### Bronchoscopy Results

Bronchoscopy results are summarized in **Table 5**. FB yielded a specific diagnosis in 162 (61.4%) cases. In 4 (1.5%) cases, FB yielded a diagnosis of malignancy consistent with the patient’s underlying disease, including leukemia in 2 (0.8%) cases and metastatic osteosarcoma in 2 (0.8%) cases.

**Table 5:** Bronchoscopy results.

Result	No. of FBs (%), n = 264
Malignant disease	4 (1.5)
Leukemia	2 (0.8)
Osteosarcoma	2 (0.8)
DAH	64 (24.2)
Lung biopsy	20 (7.6)
BOOP	1 (0.4)
Metastatic high-grade osteosarcoma	1 (0.4)
Reactive pneumonitis	1 (0.4)
Granulomatous inflammation with extensive necrosis	1 (0.4)
Clinical management altered after FB	161 (61.0)
Narrowed	63 (23.9)
Expanded	98 (37.1)

FB, flexible bronchoscopy; DAH, diffuse alveolar hemorrhage; BOOP, bronchiolitis obliterans organizing pneumonia.

In 64 (24.2%) FB cases, diffuse alveolar hemorrhage, defined as greater than 20% hemosiderin-laden macrophages present in BAL fluid, was present. Fourteen BALs returned fluid described as bloody.

The most common organisms retrieved from BAL are described in **Table 6**. In 157 (59.5%) FB cases, BAL yielded positive cultures with single pathogens, including 35 (13.3%) cultures with bacteria, 32 (12.1%) cultures with viruses, 37 (14.0%) cultures with fungi, 3 (1.1%) cultures with PJP, 3 (1.1%) cultures with acid-fast bacilli, and 38 (14.4%) cultures with multiple pathogens. In 6 (2.3%) FB cases, a culture was not obtained.

**Table 6:** Most common organisms retrieved from bronchoalveolar lavage samples.

Gram-positive	Gram-negative	Virus	Fungus	AFB
*Staphylococcus epidermidis*	*Stenotrophomonas maltophilia*	Parainfluenza 3	*Candida albicans*	*Mycobacterium kansaii*
*Staphylococcus aureus*	*Pseudomonas aeruginosa*	CMV	*Candida parapsilosis*	*Mycobacterium avium*
*Streptococcus pneumoniae*	*Klebsiella* sp. *(MDR)*	RSV	*Candida guillermondi*	*Mycobacterium gordonae*
	*Haemophilus* sp.	HHV6	*Candida glabrata*	
	*Proteus mirabilis*	Adenovirus	*Aspergillus terreus*	
		Influenza A	*Aspergillus niger*	
		Rhinovirus	*Pneumocystis jirovecii*	
		HSV	*Scopulariopsis*	
			*Rhizopus* sp.	
			*Cylindrocarpon*	
			*Curvularia dermatiaceous*	
			*Malbranchea* sp.	

AFB, acid-fast bacillus; CMV, cytomegalovirus; MDR, multi-drug resistant; RSV, respiratory syncytial virus; HHV6, human herpes virus 6; HSV, herpes simplex virus.

### Biopsy

Lung biopsy, which was performed in 20 (7.6%) FB cases, yielded a variety of diagnoses, including 1 (0.4%) case of bronchiolitis obliterans organizing pneumonia, 1 (0.4%) case of metastatic high-grade osteosarcoma, 1 (0.4%) case of reactive pneumonitis, and 1 (0.4%) case of granulomatous inflammation with extensive necrosis.

### Clinical Management

The clinical management was altered after FB in 161 of 233 cases (69.1%) of patients receiving empiric antimicrobial therapy. In 63 (27%) cases, antimicrobial therapy was narrowed, and in 98 (42.1%) cases, antimicrobial therapy was expanded.

### Outcomes

**Figure 1:**
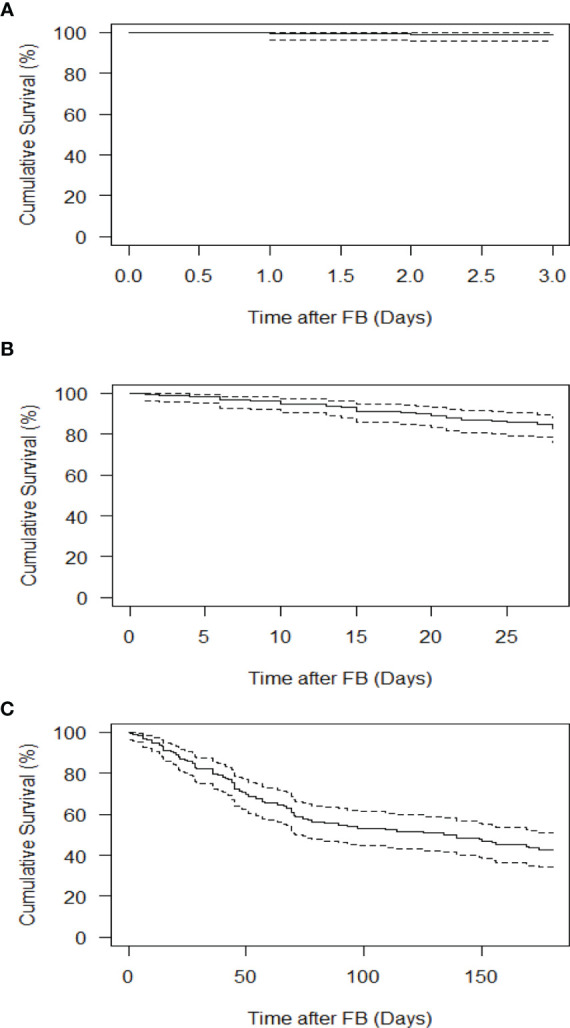
Survival of pediatric and AYA patients undergoing FB. **(A)** 3 day survival. **(B)** 28-day survival. **(C)** 180 day survival. Solid lines indicate the Kaplan-Meier survival curve, while dashed lines indicate 95% confidence intervals.

The mean duration of ICU stay was 17.4 ± 23.0 days, and the mean duration of hospitalization was 30.20 ± 31.50 days. The 3, 28 and 180 day overall survival rates were 99.0%, 86.4%, and 60.1%, respectively (**Figure 1**).

### Factors Associated With BAL Findings

**Figure 2:**
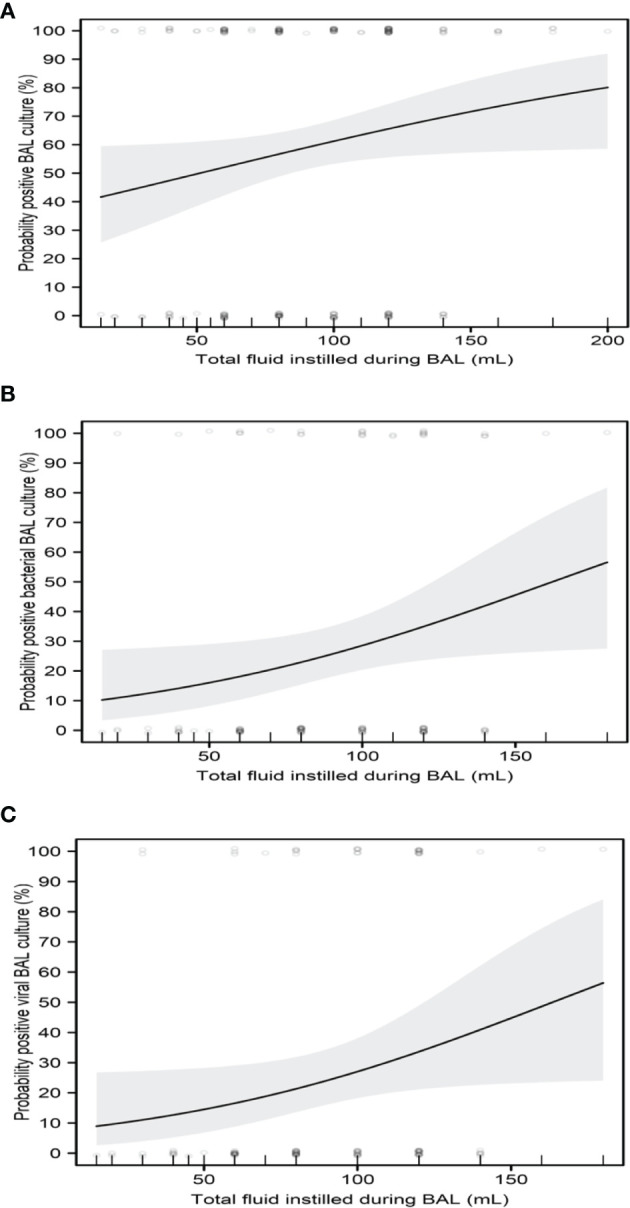
Impact of total fluid instilled during BAL on culture results. **(A)** Probability of positive BAL culture. **(B)** Probability of positive bacterial BAL culture. **(C)** Probability of positive viral BAL culture. Regression curves are shown as solid lines, bounded by shaded 95% confidence intervals. Scatterplots show raw patient measures, jittered slightly for clarity.

Increased total fluid instilled during BAL (in mL) was significantly associated with increased probabilities of positive BAL culture (p=0.042, odds ratio [OR]=1.01, 95% confidence interval [CI]=1.00-1.01), positive bacterial BAL culture (p=0.037, OR=1.01, 95% CI=1.01-1.02), and positive viral BAL culture (p=0.0496, OR=1.02, 95% CI=1.01-1.02) (**Figures 2A–C**) but not positive fungal BAL culture (p=0.38, OR=1.01, 95% CI=1.00-1.01).

**Figure 3:**
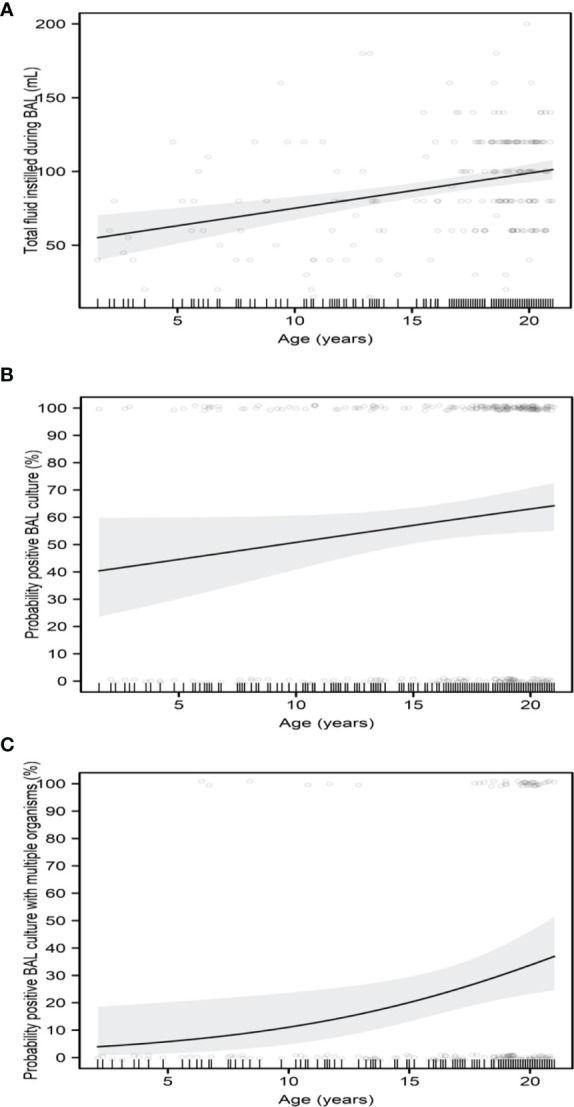
Impact of age on BAL. **(A)** Total fluid instilled during BAL. **(B)** Probability of positive BAL culture. **(C)** Probability of positive BAL culture with multiple organisms. Regression curves are shown as solid lines, bounded by shaded 95% confidence intervals. Scatterplots show raw patient measures, jittered slightly for clarity in the probability plots.

**Figure 4:**
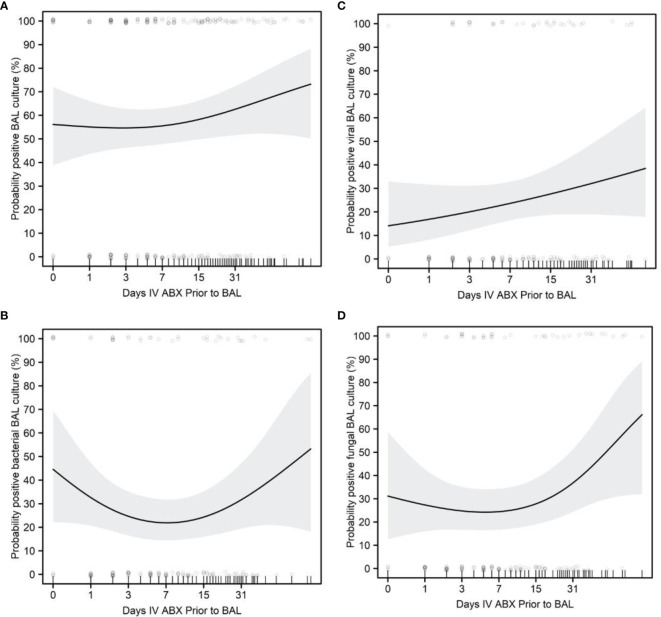
Impact of days of antibiotics and positive BAL cultures. **(A)** Probability of positive BAL culture. **(B)** Probability of positive BAL bacterial culture. **(C)** Probability of positive viral BAL culture. **(D)** Probability of positive fungal BAL culture. Regression curves are shown as solid lines, bounded by shaded 95% confidence intervals. Scatterplots show raw patient measures, jittered slightly for clarity in the probability plots.

There was a trend for older age to be associated with an increased probability of positive BAL culture (p=0.062, OR=1.05, 95% CI=1.02-1.08). Older age was significantly associated with an increased probability of positive BAL culture with multiple organisms (p=0.013, OR=1.15, 95% CI=1.09-1.22) and with a higher volume of total fluid instilled during BAL (p<0.0001, slope=2.38, 95% CI=1.39-3.36) (**Figure 3**). Our study findings did not demonstrate however a correlation between higher positive cultures for any organism from 0-7 days vs. 7-14 days and for subsets for, viral or fungal cultures with days of antibiotics and time of FB (**Figure 4**).

**Figure 5:**
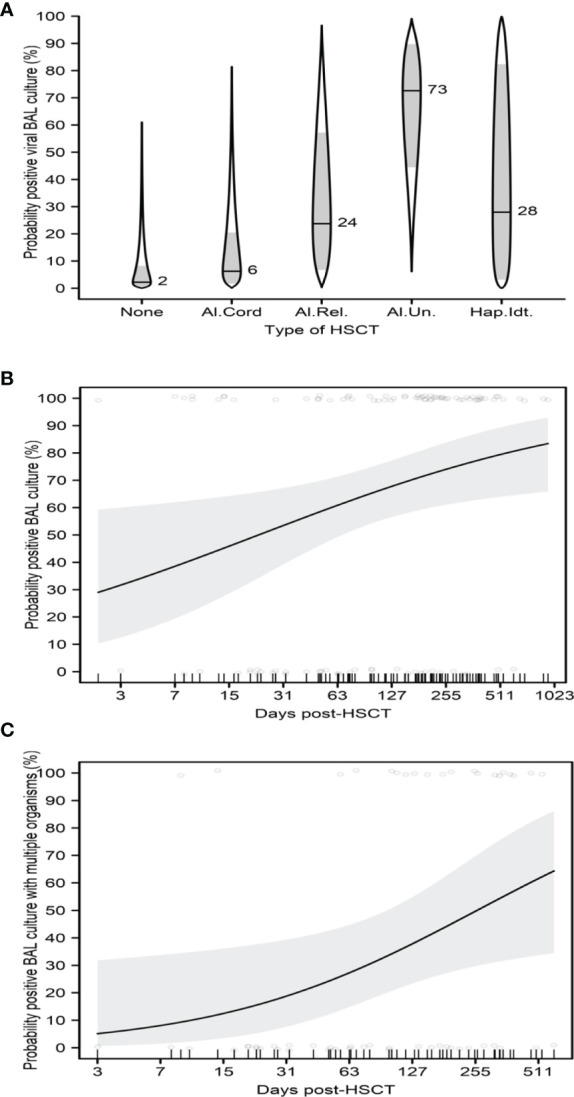
Impact of HSCT on BAL. **(A)** Probability of positive viral BAL culture with type of HSCT. **(B)** Probability of positive BAL culture with post-HSCT duration. **(C)** Probability of positive BAL culture with multiple organisms with post-HSCT duration. **(A)** shows catseye plots illustrate the normal distributions of the model-adjusted means (horizontal lines, with corresponding numeric percentage probability), with shaded +/- standard error intervals, transformed to the probability scale. In **(B, C)**, regression curves are shown as solid lines, bounded by shaded 95% confidence intervals. Scatterplots show raw patient measures, jittered slightly for clarity.

A history of HSCT was significantly associated with an increased probability of positive viral BAL culture (p=0.019, OR=16.84, 95% CI=1.59-178). Among patients with a history of HSCT, those who had received allogeneic unrelated-donor HSCT were the most likely to have a positive viral culture. Patients with a history of autologous HSCT had no positive viral BAL cultures. Longer post-HSCT duration was significantly associated with increased probabilities of positive BAL culture (p=0.017, OR=1.35, 95% CI=1.19-1.53) and positive BAL culture with multiple organisms (p=0.031, OR=1.62, 95% CI=1.3-2.02) (**Figure 5**).

An ALC <24 cells/mL was significantly associated with a longer duration of antimicrobial therapy prior to BAL (p=0.007, ratio=1.65, 95% CI=1.15-2.35). There was a trend for a lower ANC to be associated with an increased probability of positive fungal BAL culture (p=0.07, OR=0.74, 95% CI=0.62-0.87). There was also a trend for a lower AMC to be associated with an increased probability of positive fungal BAL culture (p=0.055, nonlinear estimated degrees of freedom=1.98).

## Discussion

In the current study, we describe the utility of FB with BAL among 196 pediatric cancer patients. In this, single-center review, we found that an increased total fluid volume instilled during BAL was significantly associated with increased probabilities of positive BAL culture, positive bacterial BAL culture, and positive viral BAL culture in pediatric cancer patients. Furthermore, we found that older patients were more likely to receive a large BAL instillation volume and thus had increased probabilities of a positive BAL culture and a positive BAL culture with multiple organisms. Consensus on fluid instillation, acquisition and processing in children was lacking prior to Ratjen’s (17) and Riedler’s studies (18). In 2000, The European Respiratory Society Task Force on bronchoalveolar lavage in children described a variety of protocols for BAL fluid instillation in pediatric patients (19). One approach is to instill 3 mL/kg divided into three equal aliquots for children weighing less than 20 kg and to instill 3 mL/kg in 20-mL portions for children weighing more than 20 kg (18, 20). Another approach is to adjust the BAL instillation volume according to the child’s functional residual capacity (21). Others use the method applied in adults, which is to instill 2-4 aliquots of the same volume (generally 10-20 mL), regardless of the patient’s body weight and age (22, 23). In our study, children had BAL with 3mL/kg in 10-20mL aliquots while adolescents had 2-5 aliquots of the same volume (20 mL) following best practice guidelines as referenced.

We also found a trend for both neutropenia and monocytopenia to be associated with an increased probability of positive fungal BAL culture. This is consistent with the findings of a prospective multicenter study by Villarroel et al., who found that an ANC ≤500 neutrophils/mL and AMC ≤100 monocytes/mL were each independently associated with an increased probability of invasive fungal disease (24).

In addition, we also found that the type of HSCT was associated with the probability of a positive BAL viral culture; among HSCT patients, those who underwent allogeneic unrelated-donor HSCT were the most likely to have a positive culture. This association could be explained in part by the myeloablative conditioning regimens patients receive in preparation for HSCT, whereas the predominance of allogeneic transplants may be explained by the use of immunomodulators to prevent graft-versus-host disease, each resulting in a relatively greater degree of immunosuppression (25). Our study also showed that longer post-HSCT duration was significantly associated with increased probabilities of both positive BAL culture and positive BAL culture with multiple organisms. This may be explained by the fact that after engraftment, B-cell and CD4 T-cell recover slowly; however, HSCT patients are still at risk for viral and fungal infections as well as infections caused by encapsulated bacteria (26). In addition, our findings support those of prior studies showing that BAL results influence treatment decisions (1, 27). Antimicrobial treatment was altered after more than half (69.1%) of the FB cases in the present study, with treatment narrowed in 27% of cases and treatment expanded in 42.1% of cases. In the cases where antibiotics were expanded, the most common class switch were done in patients with positive BAL cultures, worsening respiratory symptomatology or as a result of overall clinical deterioration. These included class switches from cephalosporins to carbapenems, addition of gram-positive coverage, anaerobic, antiviral, PJP and or atypical organisms’ coverage respectively. In cases where antibiotics were narrowed, the most common class switches were from carbapenems to cephalosporins or as a result of de-escalation of antiviral, PJP, antifungal coverage for patients with negative cultures, improved clinical condition or recommendation from the infectious disease consultant. Although we did not report sensitivities in the study, the data we report reflects subset of oncology and stem cell transplant patients who developed infections following standard prophylaxis strategies. While antimicrobial prophylaxis and therapies against bacterial, viral and fungal infections have improved over time, pediatric cancer and stem cell transplant patients, and perhaps, those in particular with lower respiratory tract infections may not exhibit robust responses to anti-microbial therapies in the absence of host immune defense systems (26, 28, 29).

Our findings did not demonstrate a relationship between timing of FB and diagnostic yield for infectious pathogens or between timing of FB and FB complications. This may be due to the fact that in 88.3% FBs, our subjects were receiving empiric antibiotics at the time of the FB. This is contrary to others findings, which found that as the duration of empiric antimicrobials increased, the BAL yield for positive cultures decreased (30–35).

The complication rate of FB in our study was 9.8%, with the most common complication being hypoxia (5.3%). This complication rate is lower than that described by Efrati et al. (30.6%) (36), but higher than in other studies (7, 37). A proposed pathophysiologic mechanism is that as the pathologic process involving the lung evolves, the parenchyma may become more inflamed, thereby increasing the propensity for complications such as bleeding and pneumothorax (31, 32).

Furthermore, in our study, the 3-day survival rate (99.0%) was higher than the 28-day and 180 day survival rates (86.4% and 60.1%, respectively), which suggests that patients were more likely to die from their disease than from complications related to FB.

Prior studies have also shown that FB with BAL is useful in the evaluation of pulmonary infiltrates in pediatric cancer patients. In a prospective trial of 14 pediatric patients with histories as varied as lymphoid and solid organ malignancies, solid organ transplant, and systemic lupus erythematosus, Pattishall et al. found that FB had a diagnostic yield of 71%, with the most common organisms being PJP, cytomegalovirus (CMV), *Candida*, and *Aspergillus* (27). In a retrospective analysis, Efrati et al. compared a cohort of children with cancer or primary immune deficiency (85% of whom previously received broad-spectrum antibiotics) with a control cohort of patients without a malignant diagnosis who underwent FB with BAL during the same period. A diagnosis was obtained in more than 80% of patients in both groups, and a definitive organism was detected in 53% of the patients in the cancer cohort and 18% of the patients in the control group, resulting in treatment alteration rates of 33% and 21%, respectively. Approximately 30% of the patients in the cancer cohort experienced complications, most of which were related to minor desaturations, whereas only 15% of the patients in the control group experienced complications. One cancer patient required mechanical ventilation following the procedure (36). In another retrospective study of FB with BAL in 33 patients with leukemia and pulmonary infiltrates, tracheobronchitis was identified in 51% of patients, endobronchial hypersecretion in 18%, and pulmonary hemorrhage in 21%. BAL cultures were positive in 63% of patients, revealing *Candida* in 39% of patients and *Aspergillus* in 9% of patients. There was a trend for early bronchoscopy to be associated with improved overall survival (35). In a recent review of 64 children diagnosed with leukemia, FB with BAL revealed that 56% of patients had positive microbiology results, including bacterial, viral, and fungal infections. Antimicrobial coverage was changed in more than 83% of patients. Approximately 38% of patients who underwent BAL had complications, the most common of which was hypoxia (38). Prior studies report changes in antimicrobial management after BAL (37–39) but won’t comment on prognosis and survival except for three studies in which positive and negative BAL results prompted antimicrobial changes (1, 40) but failed to show improvement in survival (36). Most of these studies, whose numbers of pediatric cancer patients ranged from 14 to 117, were relatively small compared to adult cohort studies (1, 35, 37, 41).

Our study had limitations. First, because this study was an exploratory, retrospective, single-center review, its findings may reflect technical aspects of the performance of FB and/or laboratory analysis that are unique to our institution. Second, while the data obtained *via* FB led to alteration of antimicrobial management in most cases, it is not clear whether those alterations impacted patient outcomes. The retrospective design of our study did not allow for this analysis. Lastly, the statistical analyses involved multiple testing of associations among the many variables, and no adjustment was made for multiple testing. A larger, multicenter prospective observational trial would attenuate institutional clinical practice influences and have the statistical power necessary to definitively show significant associations.

While acknowledging these limitations, our study also has several strengths. To our knowledge, this is the largest study of BAL in the pediatric oncology and HSCT population and the only BAL study in pediatric cancer patients that reports on total fluid volume instillation having an impact on BAL culture results. Additionally, our observations regarding BAL instillation volume and BAL culture positivity may help inform clinicians’ practice while performing the BAL procedure. Furthermore, the fact that BAL results altered antimicrobial treatment in more than half of the FB cases we observed also informs clinicians of the diagnostic utility of BAL when presented with pneumonias of unclear etiology in pediatric oncology and HSCT patients.

In summary, our study suggests that in pediatric oncology and HSCT patients, FB is safe, provides diagnostic and/or therapeutic benefits, and has implications for treatment decisions. Larger multicenter study evaluating the role of FB in pediatric cancer patients may further elucidate its utility for clinical care in this population.

## Data Availability Statement

The raw data supporting the conclusions of this article will be made available by the authors, upon request, without undue reservation.

## Ethics Statement

The studies involving human participants were reviewed and approved by University of Texas MD Anderson Cancer Center Institutional Review Board. Written informed consent from the participants’ legal guardian/next of kin was not required to participate in this study in accordance with the national legislation and the institutional requirements.

## Author Contributions

RM: Fiberoptic bronchoscopies, study conception, data acquisition, analysis, and manuscript writing. AA: Fiberoptic bronchoscopy assistance, data acquisition, analysis, and manuscript writing. BB: Data acquisition, analysis, and manuscript writing. CA: Statistical modeling. JC, SR, MG, and LE: Fiberoptic bronchoscopy assistance. DP and KM: Analysis. All authors contributed to the article and approved the submitted version.

## Funding

This study was supported in part by the NIH through MD Anderson’s Cancer Center Support Grant (CA16672).

## Conflict of Interest

The authors declare that the research was conducted in the absence of any commercial or financial relationships that could be construed as a potential conflict of interest.

## Publisher’s Note

All claims expressed in this article are solely those of the authors and do not necessarily represent those of their affiliated organizations, or those of the publisher, the editors and the reviewers. Any product that may be evaluated in this article, or claim that may be made by its manufacturer, is not guaranteed or endorsed by the publisher.
